# The association between the AIP and undiagnosed diabetes in ACS patients with different body mass indexes and LDL-C levels: findings from the CCC-ACS project

**DOI:** 10.1186/s12933-024-02162-w

**Published:** 2024-02-20

**Authors:** Shuwan Xu, Jun Liu, Dong Zhao, Na Yang, Yongchen Hao, Yan Zhou, Dan Zhu, Ming Cui, the CCC Investigators

**Affiliations:** 1 Department of Cardiology, Peking University Third Hospital, 49 Huayuan North Road, Haidian District, Beijing, 100191 China; 2 Department of Epidemiology and Cardiology, Beijing Anzhen Hospital, Capital Medical University, The Key Laboratory of Remodeling-Related Cardiovascular Diseases, Ministry of Education, Beijing Municipal Key Laboratory of Clinical Epidemiology, Beijing Institute of Heart, Lung and Blood Vessel Diseases, No. 2 Anzhen Road, Chaoyang District, Beijing, 100029 China

**Keywords:** AIP, Undiagnosed diabetes, ACS, Biomarker, Insulin resistance, LDL-C

## Abstract

**Background:**

The atherogenic index of plasma (AIP) has been demonstrated to be significantly associated with the incidence of prediabetes and diabetes. This study aimed to investigate the association between the AIP and undiagnosed diabetes in acute coronary syndrome (ACS) patients.

**Methods:**

Among 113,650 ACS patients treated with coronary angiography at 240 hospitals in the Improving Care for Cardiovascular Disease in China-ACS Project from 2014 to 2019, 11,221 patients with available clinical and surgical information were included. We analyzed these patients’ clinical characteristics after stratification according to AIP tertiles, body mass index (BMI) and low-density lipoprotein cholesterol (LDL-C) levels.

**Results:**

The AIP was independently associated with a greater incidence of undiagnosed diabetes. The undiagnosed diabetes was significantly greater in the T3 group than in the T1 group after adjustment for confounders [T3 OR 1.533 (1.199–1.959) p < 0.001]. This relationship was consistent within normal weight patients and patients with an LDL-C level ≥ 1.8 mmol/L. In overweight and obese patients, the AIP was significantly associated with the incidence of undiagnosed diabetes as a continuous variable after adjustment for age, sex, and BMI but not as a categorical variable. The area under the receiver operating characteristic curve (AUC) of the AIP score, triglyceride (TG) concentration, and HDL-C concentration was 0.601 (0.581–0.622; p < 0.001), 0.624 (0.603–0.645; p < 0.001), and 0.493 (0.472–0.514; p = 0.524), respectively. A nonlinear association was found between the AIP and the incidence of undiagnosed diabetes in ACS patients (p for nonlinearity < 0.001), and this trend remained consistent between males and females. The AIP may be a negative biomarker associated with undiagnosed diabetes ranging from 0.176 to 0.738.

**Conclusion:**

The AIP was significantly associated with the incidence of undiagnosed diabetes in ACS patients, especially in those with normal weight or an LDL-C level ≥ 1.8 mmol/L. A nonlinear relationship was found between the AIP and the incidence of undiagnosed diabetes, and this trend was consistent between male and female patients. The AIP may be a negative biomarker associated with undiagnosed diabetes and ranges from 0.176 to 0.738.

**Supplementary Information:**

The online version contains supplementary material available at 10.1186/s12933-024-02162-w.

## Introduction

Diabetes mellitus (DM) is a major chronic disease with serious public health and economic consequences that affects more than 400 million people worldwide [1]. Type 2 diabetes mellitus (T2DM) is the most common manifestation of DM and accounts for approximately 90–95% of all diabetes cases worldwide [2]. T2DM is characterized by gradual and asymptomatic onset and can remain undiagnosed for many years, during which various micro- and macrovascular complications may develop, progress, and remain unchecked [3]. In patients with DM, there is a two- to fourfold increased risk of developing coronary artery disease (CAD) [4]. Diabetes seems to eliminate the protective benefits of hormones against CAD in women [5]. Patients with T2DM also have hypertension, dyslipidemia, obesity, endothelial dysfunction and prothrombotic factors, called ‘metabolic syndrome’ [6, 7]. The incidence of CAD is greater among diabetic patients than among nondiabetic patients, and the mortality rate, including sudden death, is significantly greater among diabetic patients after a cardiac event than among nondiabetic patients [8]. Therefore, not only preventing the development of cardiovascular damage in DM patients but also detecting hyperglycemia progression in patients with CAD are necessary. In recent years, several studies have indicated that acute hyperglycemia, indicated by the stress–hyperglycemia ratio (SHR), is associated with poor in-hospital and long-term prognoses in acute coronary syndrome (ACS) patients [9–11]. However, in routine medical work for ACS, glycated hemoglobin (HbA1c) is not included in regular tests or oral glucose tolerance tests (OGTTs), which may limit the detection of high-risk patients.

Recently, it has been proposed that the atherogenic index of plasma (AIP), which is calculated via the logarithm of the molar concentration of triglycerides (TGs) and high-density lipoprotein cholesterol (HDL-C) [12], is associated with the incidence of prediabetes and diabetes. Prediabetes is a condition in which blood glucose parameters are above normal but below the threshold for diabetes, and it is a high-risk factor for developing diabetes [13, 14]. A retrospective cohort study included 100,069 Chinese adults and analyzed the relationship between the AIP and the risk of prediabetes; the results showed that the AIP was positively and nonlinearly associated with the risk of prediabetes after adjusting for confounding factors [15]. In another cross-sectional study among 10,099 American adults, a higher AIP was significantly associated with an increased incidence of prediabetes and diabetes, and the above relationships occurred only among women [16]. On the other hand, the AIP is reportedly correlated with the degree of insulin resistance, which can also indicate the degree of abnormal glucose metabolism [17]. These data show the relationship between the AIP and abnormal glucose metabolism. Lipid management has been widely acknowledged in routine examination and treatment of CAD patients; therefore, the AIP may help clinicians recognize those with abnormal glucose metabolism to further perform HbA1c tests and OGTTs.

However, no study has been conducted to explore the relationship between the AIP and undiagnosed DM in patients with ACS and different body mass indexes (BMIs), including underweight, normal weight, overweight, and obese individuals. Therefore, we conducted this study to determine this relationship.

## Methods

### Patient population

**Fig. 1:**
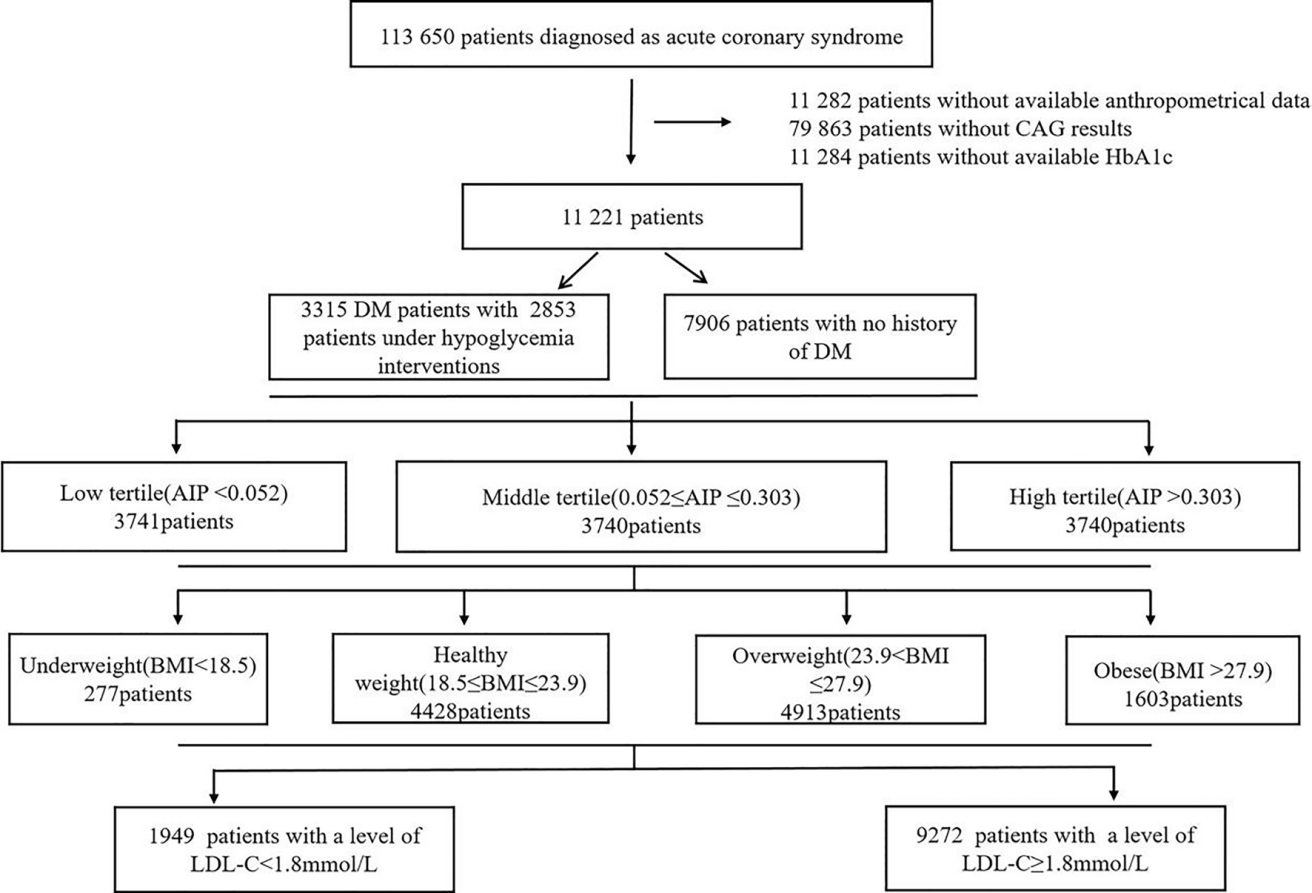
Flow diagram of the study population. *CAG* coronary angiography, *HbA1c* glycated hemoglobin, *DM* diabetes mellitus, *AIP* atherogenic index of plasma, *BMI* body mass index, *LDL-C* low-density lipoprotein cholesterol

As a collaborative initiative of the American Heart Association and the Chinese Society of Cardiology to improve the quality of care for ACS patients, the Improving Care for Cardiovascular Disease in China (CCC)-Acute Coronary Syndrome Project (CCC-ACS) is a nationwide quality improvement project involving 150 tertiary hospitals in China that was initiated in November 2014. Since 2017, the CCC-ACS Project has been extended to 82 secondary hospitals and 8 tertiary hospitals. Detailed information on the study design has been published previously [18]. The CCC-ACS was approved by the institutional review board of Beijing Anzhen Hospital, and the requirement for informed consent was waived. This study complied with the Declaration of Helsinki and is registered at the following URL: https://clinicaltrial.gov (unique identifier: NCT02306616). In this study, a total of 113,650 ACS patients from 241 hospitals in China were enrolled from 1 November 2014 to 31 December 2019. The inclusion and exclusion criteria are shown in Fig. 1*.* The final sample consisted of 11,221 ACS patients. We divided the final enrolled participants into three groups according to the tertiles of the AIP as follows: T1 group (AIP < 0.052, n = 3741); T2 group (0.052 ≤ AIP ≤ 0.303, n = 3740); and T3 group (AIP > 0.303, n = 3740).

### Study variables

The variables in this study included primary discharge information (ST-elevation myocardial infarction (STEMI), non-ST-elevation myocardial infarction, and unstable angina); demographic information (age, sex, height, weight, alcohol consumption, smoking status); medical history (hypertension, dyslipidemia, T2DM, hypoglycemia intervention, angina, myocardial infarction, percutaneous coronary intervention (PCI), and transient ischemic attack (TIA)); clinical procedure information (coronary angiography information and treatment); and laboratory test data (serum levels of total cholesterol (TC), low-density lipoprotein cholesterol (LDL-C), HDL-C, TG, fasting plasma glucose (FBG), and HbA1c).

### Definitions of the exposure and outcome variables

The exposure variable was the AIP, which was calculated using the following equation: log 10[TG (mmol/L)/HDL-C (mmol/L)] [12]. The main outcome variable in this study was undiagnosed DM, which was defined as follows: an FBG level ≥ 7.0 mmol/L and an HbA1c level ≥ 6.5%, as well as no medical history of DM or hypoglycemia treatment, including exercise, diet intervention and the use of hypoglycemia drugs. Notably, we selected this population to exclude individuals with stress hyperglycemia, which is a transient physiological response to acute disease [19]. The fasting glucose level may return to the normal concentration after the critical stress factor is removed. Stress hyperglycemia generally refers to transient hyperglycemia during illness and is usually restricted to patients without previous evidence of diabetes. Patients with a well-controlled HbA1c level whose glucose concentration was higher than the threshold defined for DM were diagnosed with stress hyperglycemia [19].

### Definitions of other variables

BMI was calculated by the following formula: weight (kg)/height (m)^2^. Participants with a BMI < 18.5 kg/m^2^, 18.5–23.9 kg/m^2^, 24.0–27.9 kg/m^2^, or ≥ 28.0 kg/m^2^ were defined as underweight, normal weight, overweight or obese, respectively. Participants were divided into two subgroups based on LDL-C levels (LDL-C level < 1.8 mmol/L and LDL-C level ≥ 1.8 mmol/L). Hypertension was defined as a history of hypertension, receipt of antihypertensive therapy, a systolic blood pressure ≥ 140 mmHg or a diastolic blood pressure ≥ 90 mmHg on admission. According to the American Diabetes Association criteria, DM was diagnosed when participants had the following conditions: an FBG ≥ 7.0 mmol/L, a 2-h plasma glucose ≥ 11.1 mmol/L according to the oral glucose tolerance test, an HbA1c ≥ 6.5%, or a history of diabetes combined with hypoglycemia intervention.

### Statistical analysis

The baseline characteristics and clinical outcomes were compared among the three groups based on the AIP. Continuous variables are expressed as means ± standard deviations if they were normally distributed and as medians (interquartile ranges) if they were not, and categorical variables are expressed as counts and percentages. One-way ANOVA and the Kruskal–Wallis rank‐sum test were used to compare differences as appropriate. Fisher's exact test and the chi‐square test were used to compare between‐group differences in categorical variables. To analyze the association between the AIP and undiagnosed DM, odds ratios (ORs) and 95% confidence intervals (CIs) were calculated using univariate and multivariate logistic regression analyses. In univariate logistic regression analysis, we selected all the significant factors to establish the models used for multivariate logistic regression analysis. We established three models to explore the association between the AIP and undiagnosed DM, which are described in the Results section. Model a was unadjusted. Model b was adjusted for age, sex, and BMI. Model c was adjusted for all the significant factors in the univariate logistic regression analysis. Notably, regarding the important effect of sex on the development of ACS, we included sex in Model b and Model c, although it was not significant in the univariate logistic regression analysis. In addition, we explored and compared the diagnostic values of the AIP, TG concentration, and HDL-C concentration for undiagnosed DM by receiver operating characteristic (ROC) curve analysis via the area under the ROC curve (AUC) and Youden’s index. To determine the association between the AIP and undiagnosed DM within different specific populations, we stratified patients into four groups according to BMI (underweight, normal weight, overweight, and obese) and two groups according to whether the serum LDL-C level was < 1.8 mmol/L. We subsequently performed an interaction analysis between the AIP and sex, BMI subgroup, and LDL-C subgroup via univariate analysis of the general linear model. In these subgroups, we studied the relationships between the AIP and the incidence of undiagnosed DM by binary logistic regressions in the models described above. The nonlinear association between the AIP and the incidence of undiagnosed DM was further evaluated by restricted cubic splines (RCSs) via the logistic regression model described above. We selected four knots, which were recommended for a better fit of the model and guaranteed accuracy. Moreover, we also performed RCS analysis of males and females in Model c. SPSS (version 27.0, SPSS, Inc., Chicago, IL, USA) and R (version 4.3.1) were used to perform the statistical analyses. P < 0.05 indicated statistical significance.

## Results

### Clinical characteristics

The baseline characteristics were significantly different across the three groups (Table 1). Patients in the T3 group were younger, more often male, had a higher BMI, were more likely to smoke and consume alcohol, and were more likely to have dyslipidemia hypertension, DM and hypoglycemia intervention; however, they were less likely to have TIA than were those in the T1 group. Moreover, they were more likely to be overweight or obese. According to the laboratory test results, patients in the T3 group had higher FBG, HbA1c, TC, LDL-C, and TG levels and lower HDL-C levels than did those in the T1 group. Notably, there was no significant difference in the medical history of myocardial infarction, angina, or PCI among the three groups. There were significantly more undiagnosed DM patients in the T3 group.

**Table 1:** Demographic and clinical characteristics of the study population according to the AIP

	Low tertile (n = 3741)	Middle tertile (n = 3740)	High tertile (n = 3740)	P value
Sociodemographic characteristics				
Age (years)	66 ± 11	63 ± 11	59 ± 12	< 0.001
Male sex	2748 (73.5%)	2822 (75.5%)	2971 (79.4%)	< 0.001
BMI	23.8 ± 3.3	24.8 ± 3.3	25.4 ± 3.4	< 0.001
Smokes	1690 (45.2%)	1821 (48.7%)	2017 (53.9%)	< 0.001
Drinks alcohol	706 (18.9%)	733 (19.6%)	829 (22.2%)	< 0.001
Medical history				
Hypertension	1959 (52.4%)	2080 (55.6%)	2159 (57.7%)	< 0.001
Dyslipidemia	307 (8.2%)	423 (11.3%)	520 (13.9%)	< 0.001
DM	874 (23.3%)	1127 (30.1%)	1314 (35.1%)	< 0.001
Hypoglycemia intervention	765(26.8%)	971(34.0%)	1117 (39.1%)	< 0.001
Angina	971 (26.0%)	935 (25.0%)	978 (26.1%)	0.476
Myocardial infarction	335 (9.0%)	339 (9.1%)	362 (9.7%)	0.506
PCI	438 (11.7%)	440 (11.8%)	437 (11.7%)	0.994
TIA	289 (7.7%)	285 (7.6%)	220 (5.9%)	0.002
Body mass index status				
Underweight	160 (4.3%)	59 (1.6%)	58 (1.6%)	< 0.001
Healthy weight	1819 (48.6%)	1432 (38.3%)	1177 (31.5%)	< 0.001
Overweight	1437 (38.4%)	1704 (45.6%)	1772 (47.4%)	< 0.001
Obese	325 (8.7%)	545 (14.6%)	733 (19.6%)	< 0.001
Laboratory tests				
FBG (mmol/L)	5.8 (5.0–7.4)	6.0 (5.1–7.8)	6.4 (5.2–8.5)	< 0.001
HbA1c (%)	5.9 (5.5–6.6)	6.1 (5.6–7.2)	6.4 (5.7–7.8)	< 0.001
TC (mmol/L)	4.3 ± 1.1	4.4 ± 1.2	4.6 ± 1.4	< 0.001
LDL-C (mmol/L)	2.6 ± 1.0	2.8 ± 1.0	2.8 ± 1.0	< 0.001
TG (mmol/L)	0.9 (0.7–1.1)	1.5 (1.3–1.8)	2.7 (2.1–3.6)	< 0.001
HDL-C (mmol/L)	1.2 (1.1–1.4)	1.0 (0.9–1.2)	0.9 (0.8–1.0)	< 0.001
AIP	− 0.143 ± 0.158	0.177 ± 0.071	0.524 ± 0.191	< 0.001
Outcome				
Undiagnosed DM	170 (22.4%)	233 (30.8%)	353 (46.6%)	< 0.001

*AIP* atherogenic index of plasma,* BMI* body mass index, *DM* diabetes mellitus, *PCI* percutaneous coronary intervention, *TIA* transient ischemic attack, *FBG* fasting blood glucose, *HbA1c* glycated hemoglobin, *TC* total cholesterol, *LDL-C* low-density lipoprotein cholesterol, *HDL-C* high-density lipoprotein cholesterol

### Association between the AIP and undiagnosed DM

As presented in Table 2, we performed univariate and multivariate logistic regression analyses to explore the potential factors related to the incidence of undiagnosed DM. The univariate analysis suggested that age, BMI, history of hypertension, dyslipidemia, angina, myocardial infarction, PCI, FBG, HbA1c, TC, LDL-C, and TG levels and the AIP were significantly associated with undiagnosed DM. After multivariate analysis, it was shown that BMI, medical history of hypertension and dyslipidemia and FBG levels, TC levels, and AIP values were still significantly related to the incidence of undiagnosed DM. Based on these explorations, we constructed three models, which were described previously.

**Table 2:** Associations between relevant factors and undiagnosed DM

Variables	Undiagnosed DM			Undiagnosed DM		
	OR	95% CI	P value	OR	95% CI	P value
Age	0.985	0.979–0.991	< 0.001			
Sex						
Male	Reference					
Female	1.011	0.851–1.202	0.899			
BMI	1.041	1.020–1.063	< 0.001	1.033	1.009–1.057	0.006
Smokes						
No	Reference					
Yes	1.026	0.885–1.189	0.731			
Drinks alcohol						
No	Reference					
Yes	0.916	0.759–1.105	0.358			
Hypertension						
No	Reference			Reference		
Yes	0.798	0.689–0.925	0.003	0.788	0.670–0.927	0.004
Dyslipidemia						
No	Reference			Reference		
Yes	0.463	0.338–0.633	< 0.001	0.458	0.329–0.638	< 0.001
Angina						
No	Reference					
Yes	0.682	0.566–0.820	< 0.001			
Myocardial infarction						
No	Reference					
Yes	0.546	0.396–0.753	< 0.001			
PCI						
No	Reference					
Yes	0.561	0.423–0.745	< 0.001			
TIA						
No	Reference					
Yes	0.821	0.602–1.120	0.213			
FBG	1.278	1.253–1.303	< 0.001	1.272	1.247–1.298	< 0.001
HbA1c	1.053	1.029–1.078	< 0.001			
TC	1.284	1.215–1.358	< 0.001	1.202	1.077–1.343	0.001
LDL-C	1.218	1.134–1.308	< 0.001			
TG	1.194	1.153–1.236	< 0.001			
HDL-C	1.083	0.915–1.281	0.354			
AIP	2.986	2.369–3.764	< 0.001	1.981	1.287–3.050	0.002

*DM* diabetes mellitus, *BMI* body mass index, *PCI* percutaneous coronary intervention, *TIA* transient ischemic attack, *FBG* fasting blood glucose, *HbA1c* glycated hemoglobin, *TC* total cholesterol, *LDL-C* low-density lipoprotein cholesterol, *HDL-C* high-density lipoprotein cholesterol*, AIP* atherogenic index of plasma, *OR* odds ratio, *CI* confidence interval

As shown in Table 3, The AIP was significantly related to undiagnosed DM as a continuous variable before and after adjustment [Model a, OR 2.986 (2.369–3.764) p < 0.001; Model b, OR 2.677 (2.102–3.409) p < 0.001; Model c, OR 1.981 (1.287–3.050) p = 0.002]. Moreover, when the AIP was considered a categorical variable, a higher AIP was associated with a greater incidence of undiagnosed DM before and after adjustment [Model a, T2 to T1 group, OR 1.396 (1.139–1.710), p = 0.001; Model b, T2 to T1 group, OR 1.332 (1.085–1.634), p = 0.006; Model c, T2 to T1 group, OR 1.222 (0.982–1.519), p = 0.072]; Model a, T3 to T1 group, OR 2.189 (1.812–2.645), p < 0.001; Model b, T3 to T1 group, OR 2.008 (1.652–2.440), p < 0.001; Model c, T3 to T1 group, OR 1.533 (1.199–1.959) p < 0.001].

**Table 3:** Associations between the AIP and undiagnosed DM

Variables	Undiagnosed DM								
	OR^a^	95% CI	P value	OR^b^	95% CI	P value	OR^c^	95% CI	P value
AIP	2.986	2.369–3.764	< 0.001	2.677	2.102–3.409	< 0.001	1.981	1.287–3.050	0.002
T1	Reference			Reference			Reference		
T2	1.396	1.139–1.710	0.001	1.332	1.085–1.634	0.006	1.222	0.982–1.519	0.072
T3	2.189	1.812–2.645	< 0.001	2.008	1.652–2.440	< 0.001	1.533	1.199–1.959	< 0.001

^a^Unadjusted model

^b^Adjusted for age, sex, and BMI

^c^Adjusted for age, sex, BMI, hypertension, dyslipidemia, angina, myocardial infarction, and PCI status, and FBG, HbA1c, TC, LDL-C, and TG levels

*AIP* atherogenic index of plasma, *DM* diabetes mellitus, *OR* odds ratio, *CI* confidence interval

**Fig. 2:**
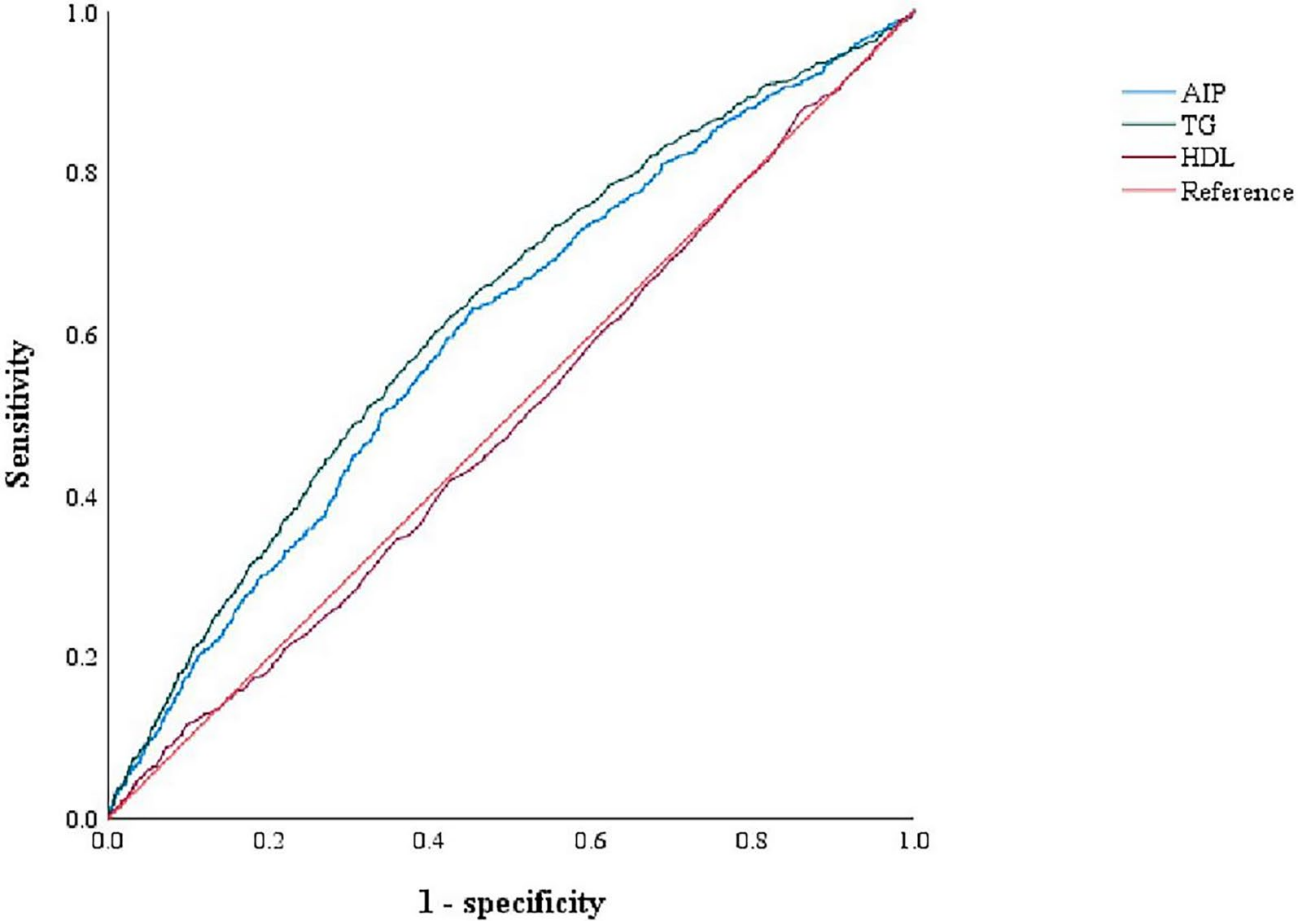
The ROC curves for AIP, TG, and HDL-C values for undiagnosed DM in ACS patients. *AIP* atherogenic index of plasma; *HDL-C* high-density lipoprotein cholesterol; *DM* diabetes mellitus; *ACS* acute coronary syndrome

The ROC curves of the AIP, TG, and HDL-C values are presented in Fig. 2. The AUC of the AIP, TG, and HDL-C values was 0.601 (0.581–0.622; p < 0.001), 0.624 (0.603–0.645; p < 0.001), and 0.493 (0.472–0.514; p = 0.524), respectively. Furthermore, we calculated the Youden index to determine the cutoff values. The best cutoff values for the AIP, TGs, and HDL-C were 0.203, 1.668, and 1.45, respectively. The corresponding sensitivities were 63.2%, 61.6%, and 11.5%, and the corresponding specificities were 54.9%, 58.1%, and 90.3%.

**Fig. 3:**
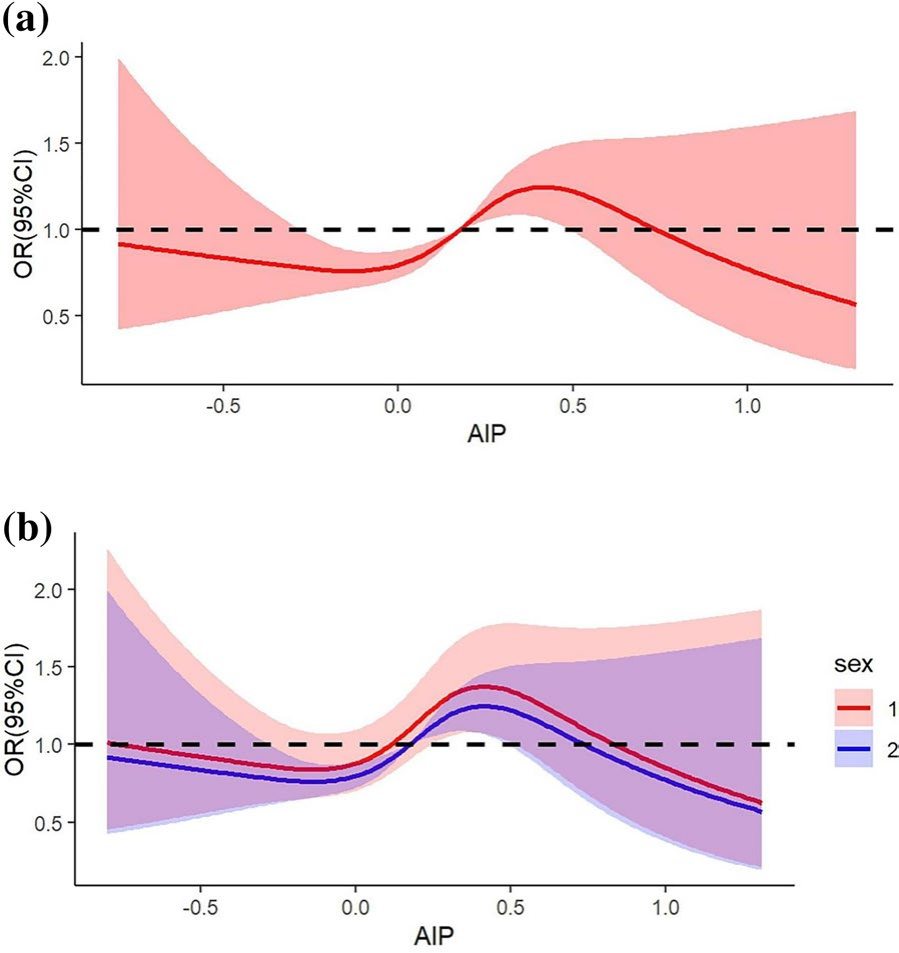
**a** Restricted cubic splines for the odds ratio of DM compared with undiagnosed DM. *DM* diabetes mellitus, *AIP* atherogenic index of plasma. **b** Sex-specific differences in the odds ratios of DM compared with undiagnosed DM according to the restricted cubic splines test.* DM* diabetes mellitus; *AIP* atherogenic index of plasma; *1* male; *2* female

In addition, we explored the nonlinear relationship between the AIP and the incidence of undiagnosed DM, as presented in Fig. 3. Figure 3a shows that after adjusting for the variables in Model c, the AIP was significantly nonlinearly associated with undiagnosed DM (p for nonlinearity < 0.001). The AIP may be a biomarker that is negatively associated with undiagnosed DM and ranges from 0.176 to 0.738. According to another cross-sectional study of 10,099 American adults, a higher AIP was significantly associated with an increased incidence of prediabetes and diabetes, and this relationship was observed only among women [16]. Thus, we performed an interaction analysis between the AIP and sex, as shown in Additional file 1: Fig. S1a; however, the results suggested that there was no significant interaction effect between the AIP and sex. We still detected sex-specific differences in the RCS, as shown in Fig. 3b. These findings suggested that the nonlinear relationship was consistent between males and females (p for nonlinearity < 0.001). For males, the AIP may be a negative biomarker that is associated with undiagnosed DM ranging from 0.112 to 0.834, while a smaller range from 0.176 to 0.738 is observed in females.

### Associations between the AIP and the incidence of undiagnosed DM according to different body mass indexes

As shown in the multivariate logistic regression analysis (Table 2), BMI was significantly positively associated with an increased incidence of undiagnosed DM. Thus, we performed an interaction analysis between the BMI subgroups described above and the AIP subgroups, as shown in Additional file 1: Fig. S1b. These results suggested that there was an interaction effect between these two factors. To further explore the effect of BMI on the AIP, we performed logistic regression analysis based on the models above (Table 4). The associations between the AIP and undiagnosed DM in underweight, normal weight, overweight and obese individuals were analyzed. There was no significant difference among underweight patients. However, a higher AIP was significantly associated with an increased incidence of undiagnosed DM in normal weight ACS patients [normal weight patients, OR^c^ 2.662 (1.187–5.967), p = 0.017]. In overweight and obese patients, the AIP, as a continuous variable, was significantly related to an increased incidence of undiagnosed DM in Model a and Model b, although this relationship disappeared in Model c [overweight patients, OR^c^ 1.333 (0.715–2.483), p = 0.365; obese patients, OR^c^ 1.819 (0.619–5.350), p = 0.277]. The positive association remained when the AIP was regarded as a categorical variable. Compared with those in the T1 group, patients in the T3 group had a greater of undiagnosed DM among normal weight patients [normal weight patients, T3 OR^c^ 1.841 (1.166–2.905), p = 0.009]. After adjustment for factors in Model b, there was a significantly greater incidence of undiagnosed DM in the overweight patients in the T3 group (overweight patients, T3 OR^b^ 1.821 (1.369–2.421), p < 0.001). However, the positive relationship between the AIP and the incidence of undiagnosed DM disappeared in obese ACS patients.

**Table 4:** Associations between the AIP and the incidence of undiagnosed DM according to different body mass statuses

Body mass status	Variables	Undiagnosed DM								
		OR^a^	95% CI	P value	OR^b^	95% CI	P value	OR^c^	95% CI	P value
Underweight (n = 277)	AIP	0.846	0.183–3.918	0.831	0.715	0.159–3.206	0.661	20.879	0255–1712.783	0.177
	T1	Reference			Reference			Reference		
	T2	1.382	0.400–4.771	0.609	1.329	0.377–4.684	0.659	2.463	0.366–16.591	0.354
	T3	1.036	0.265–4.047	0.959	0.848	0.204–3.530	0.821	1.136	0.044–29.023	0.939
Normal weight (n = 4428)	AIP	3.427	2.273–5.168	< 0.001	3.486	2.288–5.312	< 0.001	2.662	1.187–5.967	0.017
	T1	Reference			Reference			Reference		
	T2	1.891	1.361–2.628	< 0.001	1.902	1.366–2.648	< 0.001	1.562	1.087–2.245	0.016
	T3	2.560	1.850–3.542	< 0.001	2.581	1.852–3.596	< 0.001	1.841	1.166–2.905	0.009
Overweight (n = 4913)	AIP	2.879	2.035–4.072	< 0.001	2.662	1.864–3.801	< 0.001	1.333	0.715–2.483	0.365
	T1	Reference			Reference			Reference		
	T2	1.069	0.785–1.457	0.672	1.032	0.756–1.409	0.799	0.938	0.675–1.304	0.702
	T3	1.938	1.465–2.562	< 0.001	1.821	1.369–2.421	< 0.001	1.291	0.908–1.836	0.155
Obese (n = 1603)	AIP	2.183	1.244–3.833	0.007	1.893	1.053–3.403	0.033	1.819	0.619–5.350	0.277
	T1	Reference			Reference			Reference		
	T2	1.096	0.647–1.859	0.733	1.056	0.622–1.796	0.839	1.035	0.587–1.825	0.905
	T3	1.564	0.963–2.539	0.071	1.417	0.863–2.329	0.169	1.213	0.666–2.211	0.528

^a^Unadjusted model

^b^Adjusted for age, sex, and BMI

^c^Adjusted for age, sex, BMI, hypertension, dyslipidemia, angina, myocardial infarction, and PCI status, and FBG, HbA1c, TC, LDL-C, and TG levels

*AIP* atherogenic index of plasma, *DM* diabetes mellitus, *OR* odds ratio, *CI* confidence interval

### Associations between the AIP and the incidence of undiagnosed DM according to LDL-C levels

Previous studies have shown that dyslipidemia appears in the early stages of DM and is involved in disease progression throughout the entire disease course [20, 21]. LDL-C, a key cardiovascular disease marker, is often estimated by the Friedwald or Marin equation. However, many patients with elevated TG levels have suppressed LDL-C levels [22]. The AIP is calculated via the logarithm of the molar concentration of TG and HDL-C. To determine whether the relationship between the AIP and undiagnosed DM was affected by the serum LDL-C level, we divided patients into two groups based on a cutoff of 1.8 mmol/L, which is the recommended target. We subsequently performed an interaction analysis between the AIP and LDL-C subgroups, as demonstrated in Additional file 1: Fig. S1c. These results suggested that there was an interaction effect between these two factors. Therefore, we conducted binary logistic regression analysis based on the three models described above. As demonstrated in Table 5, a higher AIP was significantly associated with a higher incidence of undiagnosed DM in ACS patients with an LDL-C level ≥ 1.8 mmol/L after adjustment in Model c; however, this trend became less significant in patients with an LDL-C level < 1.8 mmol/L after adjustment in Model c (patients with an LDL-C level < 1.8 mmol/L, OR^c^ 1.523 (0.521–4.451), p = 0.442; T3 OR^c^ 1.612 (0.807–3.219), p = 0.176; patients with an LDL-C level ≥ 1.8 mmol/L, OR^c^ 2.028 (1.262–3.260), p = 0.004; T3 OR^c^ 1.507 (1.156 0.964), p = 0.002].

**Table 5:** Associations between the AIP and the incidence of undiagnosed DM according to LDL-C levels

LDL-C levels	Variables	Undiagnosed DM								
		OR^a^	95% CI	P value	OR^b^	95% CI	P value	OR^c^	95% CI	P value
LDL-C < 1.8 mmol/L (n = 1949)	AIP	3.241	1.820–5.773	< 0.001	3.304	1.795–6.082	< 0.001	1.523	0.521–4.451	0.442
	T1	Reference			Reference			Reference		
	T2	1.382	0.767–2.488	0.282	1.376	0.759–2.495	0.293	1.405	0.746–2.646	0.293
	T3	2.368	1.398–4.011	0.001	2.326	1.349–4.011	0.002	1.612	0.807–3.219	0.176
LDL-C ≥ 1.8 mmol/L (n = 9272)	AIP	2.949	2.283–3.810	< 0.001	2.627	2.011–3.432	< 0.001	2.028	1.262–3.260	0.004
	T1	Reference			Reference			Reference		
	T2	1.357	1.093–1.686	0.006	1.295	1.041–1.612	0.021	1.205	0.954–1.521	0.117
	T3	2.111	1.724–2.586	< 0.001	1.932	1.567–2.382	< 0.001	1.507	1.156–1.964	0.002

^a^Unadjusted model

^b^Adjusted for age, sex, and BMI

^c^Adjusted for age, sex, BMI, hypertension, dyslipidemia, angina, myocardial infarction, and PCI status, FBG, HbA1c, TC, LDL-C, and TG levels

*AIP* atherogenic index of plasma, *DM* diabetes mellitus, *LDL-C* low-density lipoprotein cholesterol, *OR* odds ratio, *CI* confidence interval

## Discussion

In this study, we found that the AIP was nonlinearly associated with the incidence of undiagnosed DM in ACS patients, and this relationship was consistent for both males and females In patients with a healthy weight and an LDL-C level ≥ 1.8 mmol/L, a higher AIP was significantly associated with an increased incidence of undiagnosed DM. To our knowledge, this is the first study to explore the relationship between the AIP and the incidence of undiagnosed DM in ACS patients with different BMIs, including underweight, normal weight, overweight, and obese individuals and individuals with LDL-C levels < 1.8 mmol/L.

Despite the ease of measuring plasma glucose levels, some people are unaware of their true glucose metabolism status. In this study, for the first time, we focused on people who were diagnosed with DM based on their fasting glucose concentration and HbA1c level and had no history of hypoglycemia therapy. We selected this population to exclude those with stress hyperglycemia. Owing to the effects of critical diseases such as ACS and clinical procedures, it is possible that some patients in this study may have experienced transient hospital-related hyperglycemia. Given that we lacked available FBG measurements at discharge, the diagnosis based solely on the first FBG measurement was insufficient. The level of HbA1c can provide information about glycemic control over a long period and is expected to be a multipotent and powerful cardiometabolic marker beyond the clinical setting of diabetes care [23, 24]. Notably, these patients may have been influenced by glycemia for a long time, as subtle symptoms may have gone unnoticed. The risk of adverse cardiovascular events, including CAD, has increased. Thus, focused efforts to identify patients who are at high risk of hyperglycemia-mediated harm and are likely to benefit from interventions are needed. In the present study, among 11,221 ACS patients, 756 patients had undiagnosed DM, and the AIP was strongly associated with undiagnosed DM after adjustment for covariables [OR 1.533 (1.199–1.959), p < 0.001]. However, large-scale longitudinal studies are needed to explore the relationship between the AIP and long-term outcomes in these patients.

The AIP has been proven to be significantly correlated with lipoprotein particle size and density, as well as lipoprotein peroxidation rates, and could be used as a reliable marker of plasma atherogenicity [25]. Recent research has demonstrated that the AIP is a superior predictor of plasma atherogenicity compared to isolated lipid values and that the AIP is strongly correlated with an increased incidence of subclinical or symptomatic CAD [26–28]. Lipid-lowering medication, including statins, is the basis for the treatment of CAD. Several cumulative studies suggest that statins are associated with an increased risk of new-onset DM [29–32]. However, it was also proposed that the cardiovascular benefits of the use of statins exceed the risk of complications, and the low incidence of new-onset DM should not hinder the wide application of statins among CAD patients. Moreover, lifestyle changes, including healthy diet and exercise, and timely observation of blood glucose levels are recommended to reduce the risk of diabetes once statins are used [33].

However, the mechanism underlying these results is unclear. There are likely multiple plausible explanations for these findings. One possible explanation is that atherogenicity may be a major factor, as the AIP is calculated from TG and HDL-C levels. When the plasma TG concentration is greater, LDL-C particles tend to be smaller, denser, more easily oxidized, and more prone to entering the subintima, thereby increasing their atherogenic potential. Previous research has demonstrated a positive correlation between the AIP and the particle size of small, dense LDLs, indicating that the AIP may serve as a reliable marker of atherogenesis [34]. LDL-C is the principal intervention target in the risk management of atherosclerotic cardiovascular disease (ASCVD) [35]. Undiagnosed diabetes may result in incorrect risk stratification for patients receiving lipid-lowering therapy. According to a cross-sectional analysis of the National Health and Nutrition Examination Survey (NHANES) (2005–2010), improved screening for diabetes and a reduced incidence of undiagnosed diabetes may help to identify individuals requiring more intensive LDL-C reductions [36]. In our present study, we divided ACS patients into two groups based on their serum LDL-C level (LDL-C < 1.8 mmol/L). The AIP was suggested to be positively associated with the increased incidence of undiagnosed DM, particularly in patients with an LDL-C level ≥ 1.8 mmol/L.

Moreover, several studies have demonstrated significant associations between an elevated AIP and insulin resistance, which is associated with increased susceptibility to diabetes [17, 37, 38]. High plasma TG levels reduce the number and activity of insulin receptors on adipocytes and prevent insulin from binding to receptors by competing with glucose to enter cells, leading to abnormal glucose metabolism, while lower HDL levels also lead to decreased insulin secretion and sensitivity [39–41]. On the other hand, abnormal blood lipid levels may cause insulin resistance (IR) by causing inflammation, endoplasmic reticulum stress and lipotoxicity [42, 43]. A Chinese population-based cross-sectional study demonstrated that a higher AIP was positively and strongly associated with obesity, suggesting that the AIP is a novel and better biomarker associated with obesity [44]. Obesity is also an established risk factor for insulin resistance and diabetes. In our study, we also stratified ACS patients into four groups according to their BMI: underweight, normal weight, overweight, and obese. However, in our study, we found that the AIP was positively associated with an increased incidence of undiagnosed DM only in individuals who were normal weight after we adjusted for all the covariates, and this association was not as strong as that in overweight and obese patients and was not present in underweight patients. More evidence based on underweight, overweight and obese participants is needed.

There are several limitations that require consideration in the current study. First, due to the observational nature of this study, despite adjusting for potential risk factors, residual or unmeasured confounding variables may still exist. Second, we did not carry out follow-up, and the outcomes outside the hospital were not clear. Adequately powerful prospective cohort studies will be necessary to confirm the relationship between the AIP and the risk of undiagnosed DM. Third, the participants in this study were exclusively Chinese patients, and the generalizability of the findings to other ethnic groups remains uncertain. Fourth, this investigation solely examined the baseline AIP, and longitudinal consecutive changes in the AIP during hospitalization were not analyzed. Finally, information on some lipid-lowering agents and their respective dosages, which may have influenced the results, was not available in our database.

## Conclusions

The AIP, a comprehensive index of lipid management in ACS patients, was nonlinearly associated with an increased incidence of undiagnosed DM, especially in normal-weight patients and those with an LDL-C level ≥ 1.8 mmol/L. This association was consistent between males and females. The AIP may be a biomarker that is negatively associated with undiagnosed diabetes and ranges from 0.176 to 0.738.

### Supplementary Information

**Additional file 1: Figure S1. **a The interactive analysis between the AIP and sex (p for interactive analysis=0.398). *AIP* atherogenic index of plasma. b The interactive analysis between the AIP and BMI (p for interactive analysis < 0.001). *AIP* atherogenic index of plasma; *BMI* body mass index. c The interactive analysis between the AIP and LDL-C (p for interactive analysis < 0.001). *AIP* atherogenic index of plasma; *LDL-C* low-density lipoprotein cholesterol.

## Data Availability

The data, analytical methods, and study materials will be made available for onsite audits by third parties for the purposes of reproducing the results or replicating the procedure.
