# Supplementary material for: The association between the AIP and undiagnosed diabetes in ACS patients with different body mass indexes and LDL-C levels: findings from the CCC-ACS project
Source: Cardiovasc Diabetol. 2024 Feb 20;23:77. doi: 10.1186/s12933-024-02162-w (PMC10880375; doi:10.1186/s12933-024-02162-w)
Supplement: Supplementary file 1 — Additional file 1: Figure S1. a The interactive analysis between the AIP and sex (p for interactive analysis=0.398). AIP atherogenic index of plasma. b The interactive analysis between the AIP and BMI (p for interactive analysis < 0.001). AIP atherogenic index of plasma; BMI body mass index. c The interactive analysis between the AIP and LDL-C (p for interactive analysis < 0.001). AIP atherogenic index of plasma; LDL-C low-density lipoprotein cholesterol. [file 12933_2024_2162_MOESM1_ESM.docx]

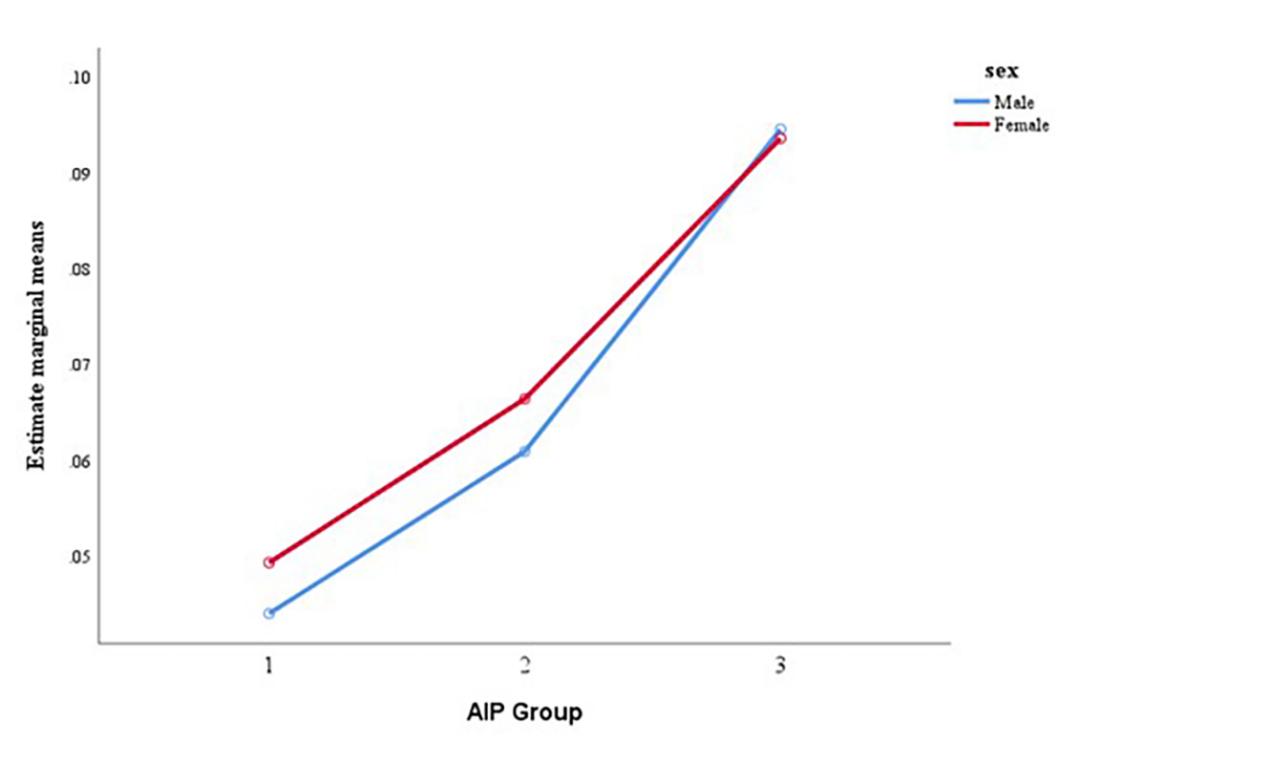


Figure S1a The interactive analysis between the AIP and sex (p for interactive analysis=0.398). *AIP* atherogenic index of plasma


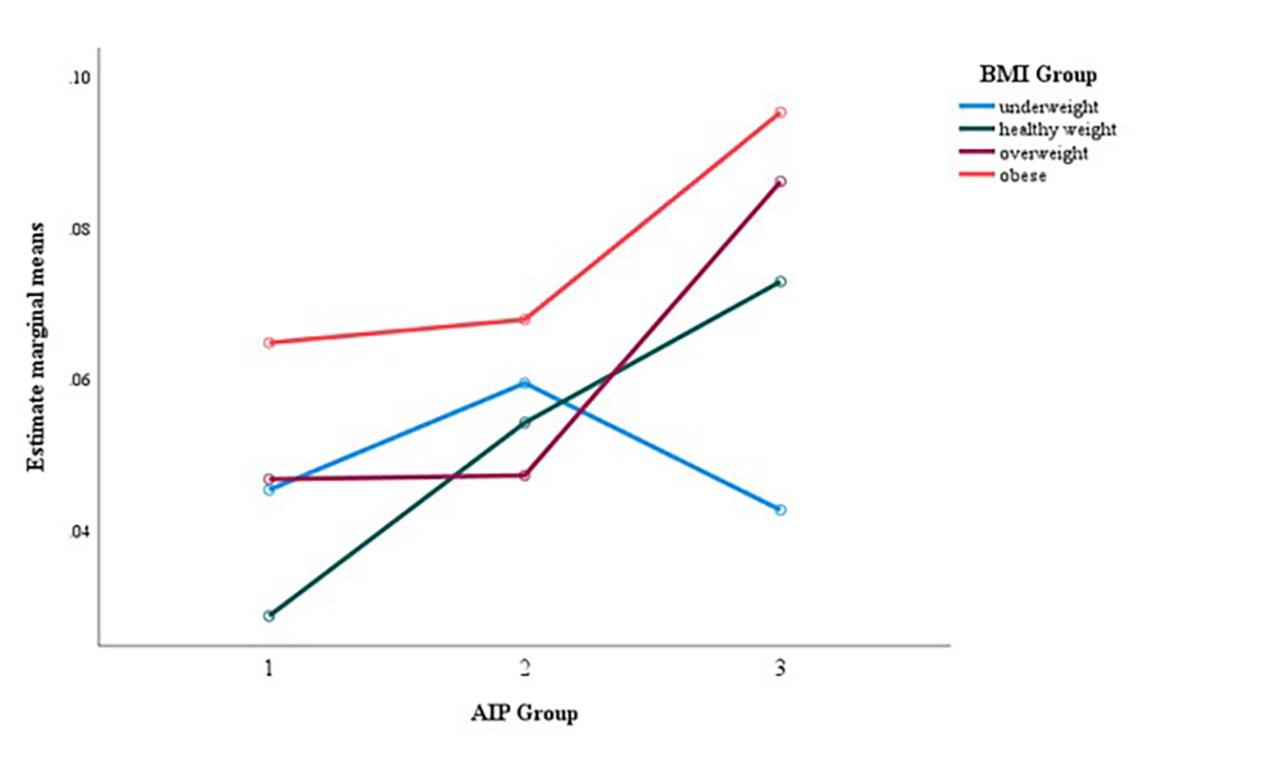


Figure S1b The interactive analysis between the AIP and BMI (p for interactive analysis<0.001). *AIP* atherogenic index of plasma; *BMI* body mass index


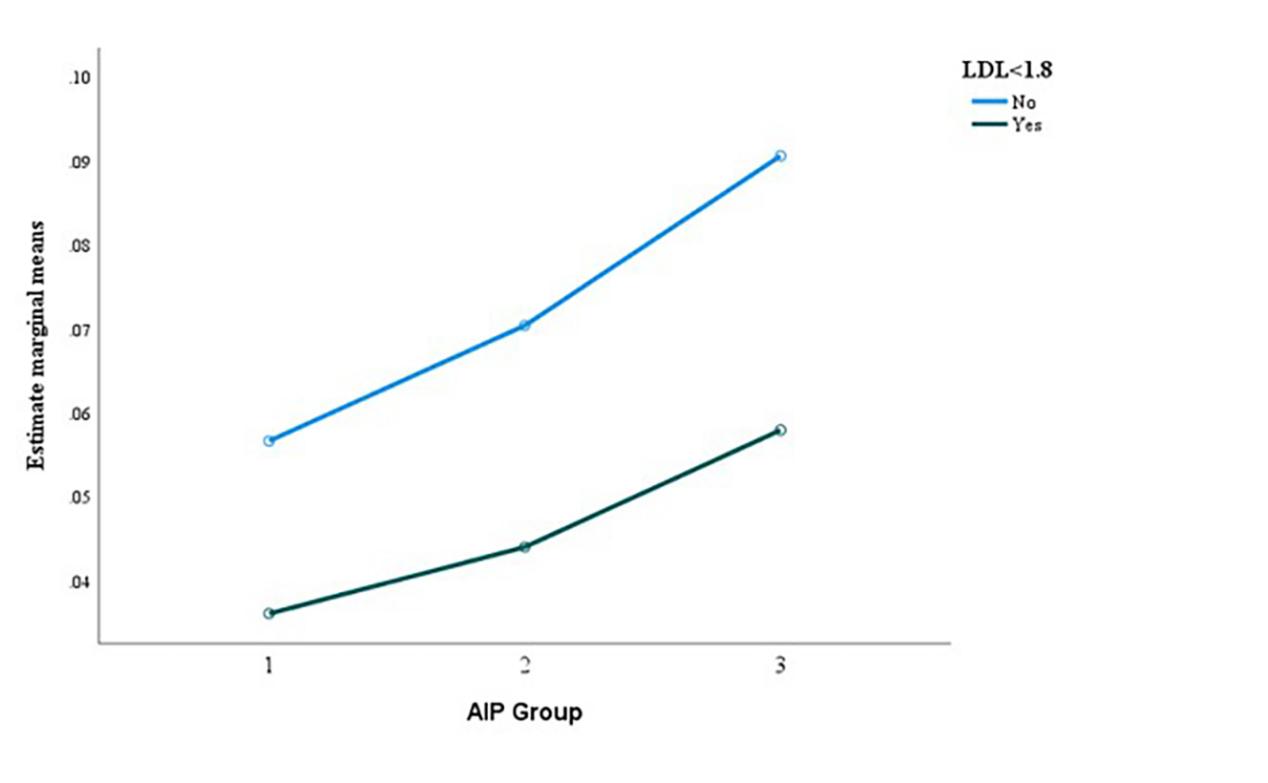


Figure S1c The interactive analysis between the AIP and LDL-C (p for interactive analysis<0.001). *AIP* atherogenic index of plasma; *LDL-C* low-density lipoprotein cholesterol
